# Financing health in sub-Saharan Africa 1990–2050: Donor dependence and expected domestic health spending

**DOI:** 10.1371/journal.pgph.0003433

**Published:** 2024-08-28

**Authors:** Angela E. Apeagyei, Brendan Lidral-Porter, Nishali Patel, Juan Solorio, Golsum Tsakalos, Yifeng Wang, Wesley Warriner, Asrat Wolde, Yingxi Zhao, Joseph L. Dieleman, Justice Nonvignon

**Affiliations:** 1 Department of Health Metrics Sciences, University of Washington School of Medicine, Institute for Health Metrics and Evaluation, Seattle, Washington, United States of America; 2 Institute for Health Metrics and Evaluation, Seattle, Washington, United States of America; 3 Nuffield Department of Medicine, University of Oxford, United Kingdom; 4 Department of Health Policy, Planning and Management, University of Ghana, Accra, Ghana; University of Hong Kong, HONG KONG

## Abstract

In 2021, global life expectancy at birth was 74 years whereas in sub-Saharan Africa it was 66 years. Yet in that same year, $92 per person was spent on health in sub- Saharan Africa, which is roughly one fifth of what the next lowest geographic region—North Africa and Middle East—spent ($379). The challenges to healthy lives in sub-Saharan Africa are many while health spending remains low. This study uses gross domestic product, government, and health spending data to give a more complete picture of the patterns of future health spending in sub-Saharan Africa. We analyzed trends in growth in gross domestic product, government health spending, development assistance for health and the prioritization of health in national spending to compare countries within sub-Saharan Africa and globally.We found that while gross domestic product was projected to increase through 2050 in sub-Saharan Africa, the share of gross domestic product that goes to health spending is only expected to increase moderately. Our exploration shows that this tepid growth is expected because the percent of overall government spending that is dedicated to health 7·2% (6·3–8·3) compared to average of 12·4% (11·7–13·2) in other regions) is expected to stay low. Even if the amount, of resources provided from donors climbs some, it is not expected to keep pace with growing economies in sub-Saharan Africa and may transition towards other global public health goods. Critically, development assistance for health provided to sub-Saharan Africa is expected to decrease in some countries, and the expected growth in government health spending might not be enough to cover this expected decline. Increases in spending with a concordant prioritization of health and the appropriate health system governance and structural reforms are critical to ensure that people who live in sub-Saharan Africa are not left behind.

## Introduction

While high spending on health alone does not guarantee good health outcomes [1], spending on health is necessary to put in place the system for health delivery–namely the health facilities, health workers, drugs and other essential health resources [2–4]. Therefore, examining how much has been and how much is expected to be spent on health is useful for planning and for identifying potential financing gaps that need to be filled.

Health spending in sub-Saharan Africa is significantly lower than any other region in the world. In 2021, $92 per person was spent on health, which is roughly one fifth of what the next lowest region—North Africa and Middle East spent on health ($379). Moreover, the projected growth in health spending is concerning for sub–Saharan Africa–per person health spending is projected to be $86·3 (60·1–111·9) in 2050, with a flattened annualized growth rate of 0·7% (0·5–0·9)) compared to a global average of 1·0% (0·6–1·5) [5].

In addition to the low health spending, sub-Saharan Africa faces significant disease burden with both communicable and non-communicable diseases reporting high mortality levels. In 2021, the number of deaths due to communicable diseases reached 4.29 million, marking a 23.8% rise in deaths since 2010, whereas deaths from non-communicable diseases was 2.92 million in the same period [6].

Furthermore, while the health toll of the COVID-19 pandemic was moderate in the region, the economic toll due to lockdowns and other mitigation strategies was enormous [7]. In several African countries, mounting debt obligations, inflationary and exchange rate pressures continue to present a threat to economic growth [8]. Specifically, 2023 saw sub-Saharan African economies grapple with inflationary shock following global events such as Russia’s war in Ukraine, higher interest rates, reduced international demand, and ongoing exchange rate pressures. This complex backdrop has led to a fall in growth to 3.3 percent in 2023, with a cautious outlook for recovery in 2024. Political instability and external economic risks, including the potential slowdown in major economies like China, add layers of uncertainty to this recovery trajectory [9]. Despite these challenges, the region is anticipated to rebound, with growth increasing in most countries, particularly those less reliant on natural resources. However, that recovery is fragile and contingent on countries been able to maintain the appropriate policy stance to manage inflation, exchange rate fluctuations and debt obligation pressures. This nuanced economic landscape underscores the need for a multifaceted approach to health financing and economic recovery in sub-Saharan Africa [9–12].

The objective of this study is to examine historic and future health spending estimates with a focus on sub–Saharan Africa to highlight the expected trends, characterize expected spending patterns and examine potential drivers of the expected spending patterns. The analysis reviews sources of spending in sub-Saharan Africa, government and development assistance from 1990 through 2050. The expected health spending scenarios are analyzed considering macroeconomic projections to provide the broader context for which health spending will occur. Such analyses are valuable because the findings help policy makers understand future health spending patterns to be able to adjust actions or make changes in policy that ensures that desired future outcomes are realized.

## Methods

We utilized data from the following four databases: Development Assistance for Health [13], Global Health Spending [14], Global Expected Health Spending [5] and Gross Domestic Spending (GDP) per capita [15], all produced by the Global Burden of Disease Health Financing Collaborator Network. Notably, for DAH, we not only sourced data from established databases like the OECD’s Creditor Reporting System (CRS) and prominent organizations such as the World Bank, the Global Fund, CEPI, and philanthropic entities like the Bill & Melinda Gates Foundation and Susan Thompson Buffet Foundation, but we also obtained valuable data directly through correspondence with some of the international development organizations. The DAH database reports the financial and in-kind resources transferred through international development agencies to low- and middle-income countries with the primary aim of maintaining or improving health [16]. The database includes reported contributions from 1990 through 2022. We generated the dataset utilizing project level information on donor contributions reported in online databases by major international development agencies such as the Organization for Economic Cooperation and Development, the Global Fund to fight AIDS, Tuberculosis and Malaria, UN agencies and the Bill and Melinda Gates Foundation as well as data from annual reports, financial statements and budget reports. It maps contributions from originating sources through disbursing agencies (known as channels) to health focus areas, program areas and recipient entities. It also includes an estimate of donor contributions for the most recent full year based on data provided in budget documents. The estimation accounted for double counting by relying on data from annual reports and financial reports on income sources to exclude resource flows that are moved from one agency to another prior to disbursing. This process ensures that each funding flow is counted only once. A detailed explanation of how these estimates were generated is extensively documented elsewhere [13, 16].

The Global Health Spending database covers spending on health from four sources mutually exclusive and collective exhaustive financing sources: government, development assistance, out of pocket spending and pre-paid private. The health spending data reported in this database is from 1995 through 2021. Excluding the DAH data, we relied on reported disaggregated health spending data in the WHO’s Global Health Expenditure Database (GHED) for input data to this database. Government health spending captures spending on health from government revenues exclusively, out of pocket spending covers direct household spending on health and pre-paid private captured private insurance and/or NGO prepaid services. We conducted a qualitative assessment of each of the extracted data points based on metadata provided in the GHED and include only data points based on reliable data sources. To fill missingness, we leveraged a Spatio-temporal Gaussian Process Regression (ST-GPR). ST-GPR is a modeling approach that leverages available data, data from neighboring countries and years, and specified covariates to generate estimates of spending where estimates are missing. It also generates estimates of uncertainty. These uncertainty intervals are largest when there is no input data or when the available input data are contradictory. An extensive explanation of the process undertaken to generate these estimates is reported elsewhere [16].

The Expected Health Spending database provides projected health spending data from 2022 through 2050. To generate these estimates, we relied on input data from diverse sources including the Global Health Spending database described above. An ensemble modeling technique was used to generate the estimates of future health spending, which included multiple submodels spanning a number of predictors and model specifications. We utilized out-of-sample validation to select the best performing submodels, so that each estimate was the mean of 500 other projected estimates from different submodels. The projections include different types of uncertainty, including modeling uncertainty, data uncertainty, and uncertainty of the parameter estimates. The forecasting process was sequential and includes forecasts of the growth in gross domestic product (GDP) per working age adults, general government spending debt as a proportion of general government spending, official development assistance as a proportion of general government spending, and modeled availability of future DAH donated and received. A detailed explanation of how these estimates were generated is extensively documented elsewhere [16].

Lastly, the Gross Domestic Product per capita database reports estimates of Gross Domestic Product from 1960 through 2050. The historic estimates are based on input data from a diverse set of sources and employ methods and approach detailed extensively elsewhere to generate the projected gross domestic product [15, 16].

We extracted these health spending estimates to provide insight into the trajectory of expected future health spending in sub-Saharan Africa. We completed these analyses on the extracted data. First, we calculated the change in gross domestic product across countries to examine historic and future changes in the macroeconomic context of countries in sub-Saharan Africa. We then calculated and analyzed historic and prospective rates of growth for both donor and government health spending to assess past and future availability of resources. Additionally, we assessed how the share of gross domestic product that is health spending has grown historically, how it is expected to evolve in the future and also how the different sources of financing will evolve, to highlight resource availability for the health sector given the broader macroeconomic context. Lastly, we examined past and prospective trends in the share of gross domestic spending that is general government spending as well as the share of general government spending that is spent on health to understand the drivers of the changes in government health spending. All analyses were completed with R version 4.2.0 (2022-12-23).

## Results

**Fig 1:**
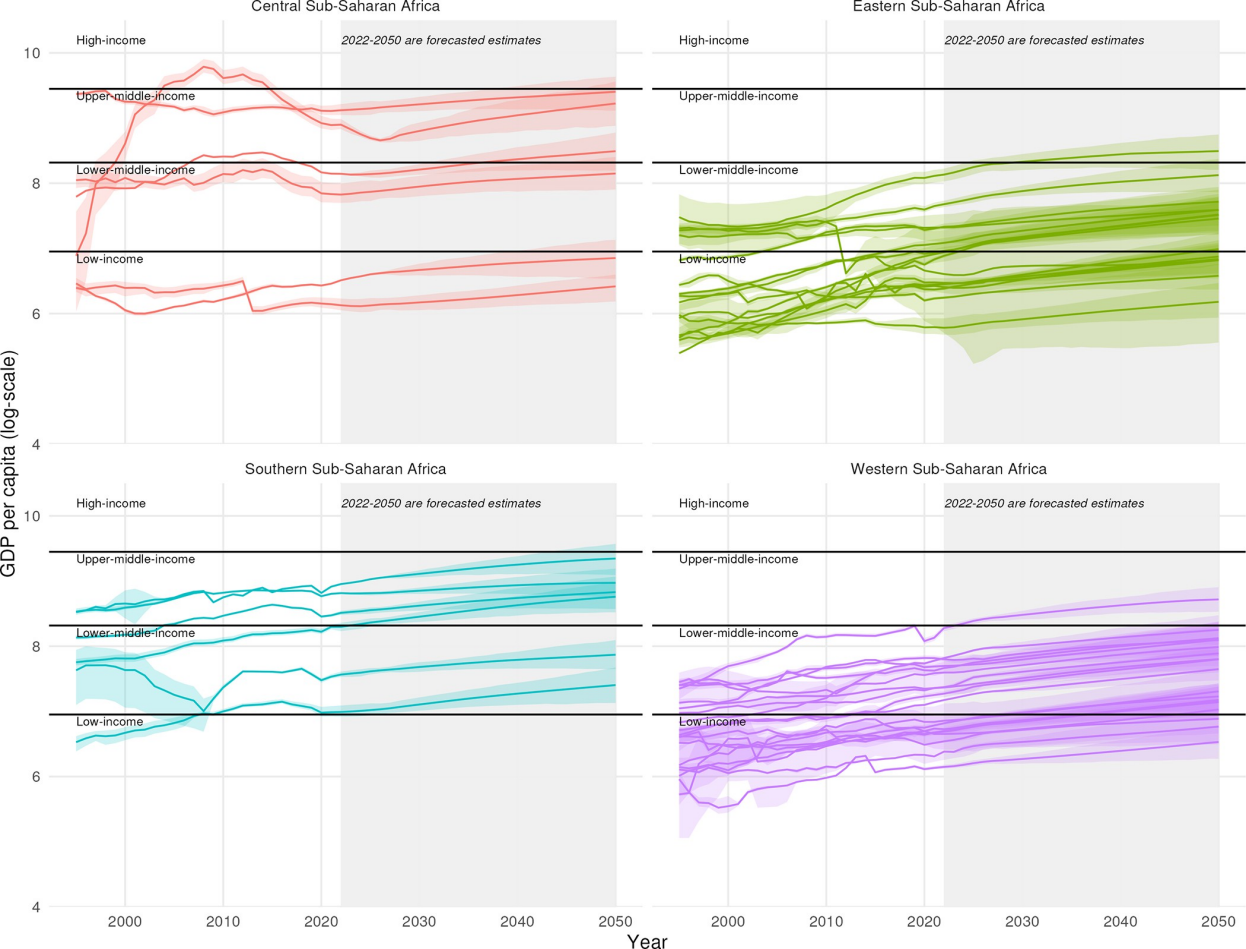
Changes in GDP per capita across World Bank income categories in four sub-regions of Sub-Saharan Africa, 1995–2050. Income groups are based on World Bank classifications for 2021. Estimates for 2022 to 2050 are forecasted. Central sub-Saharan Africa includes Angola, Central African Republic, Congo, Democratic Republic of the Congo, Equatorial Guinea, and Gabon. Eastern sub-Saharan Africa includes Burundi, Comoros, Djibouti, Eritrea, Ethiopia, Kenya, Madagascar, Malawi, Mozambique, Rwanda, Somalia, South Sudan, Uganda, United Republic of Tanzania, and Zambia. Southern sub-Saharan Africa includes Botswana, Eswatini, Lesotho, Namibia, South Africa, and Zimbabwe. Western sub-Saharan Africa includes Benin, Burkina Faso, Cabo Verde, Cameroon, Chad, Côte d’Ivoire, Gambia, Ghana, Guinea, Guinea-Bissau, Liberia, Mali, Mauritania, Niger, Nigeria, Sao Tome and Principe, Senegal, Sierra Leone, and Togo.

While sub–Saharan African countries have experienced economic growth and are expected to grow in the future, very few countries are expected to experience growth that will push them into World Bank higher-income categories. Fig 1 traces the growth in GDP per person across the four sub-regions of sub-Saharan Africa from 1995 through 2050. Across the sub-regions in Africa, there are a few increases in GDP per capita that fall within the upper-middle- and high-income categories and no country is expected to transition to high-income category. While many countries in Western and Eastern sub-Saharan Africa are low-income countries with historical and projected changes in GDP growth suggesting they will remain in the low-income country, 14 countries are projected to transition to lower middle-income based on expected GDP per capita levels. Historically, most of the countries across sub-Saharan Africa have been categorized as low and lower middle-income categories and the forecasted estimates of changes in GDP including uncertainty highlight that many countries will also continue to remain in the low and lower middle-income categories going forward. The expected trends are driven by the historic growth rates observed in GDP, the impact of rising interest rates and increasing debt repayments in some countries.

**Fig 2:**
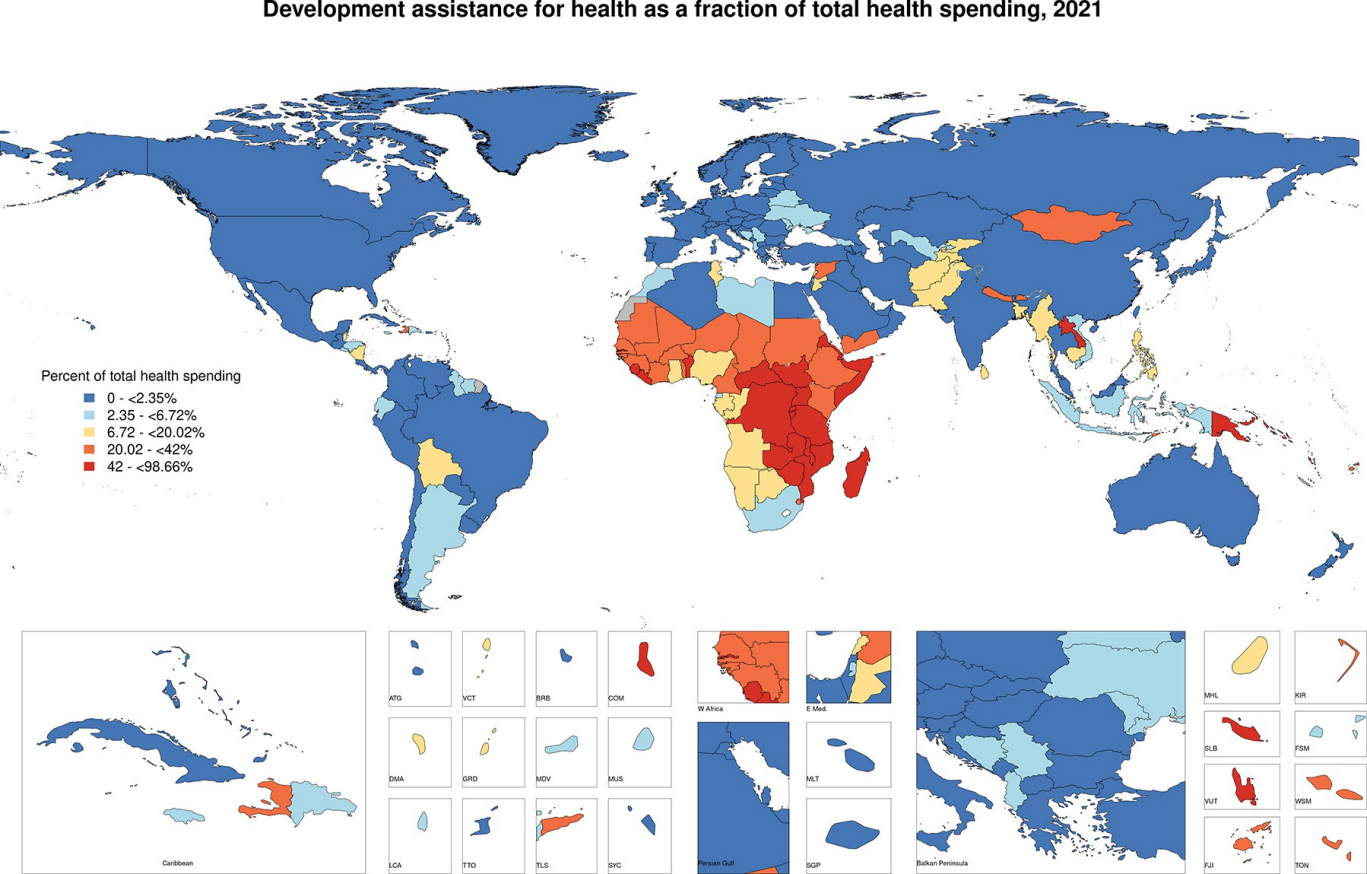
Proportion of health spending that is development assistance, 2021.

Furthermore, globally sub-Saharan Africa is the region within which development assistance represents the largest source of health spending. This is highlighted in the map presented in Fig 2 which shows the share of health spending from donors across countries. In 2021, half of the countries in Sub-Saharan Africa, specifically 23 out of 46, relied on external financing for more than a third of their health expenditures.

**Fig 3:**
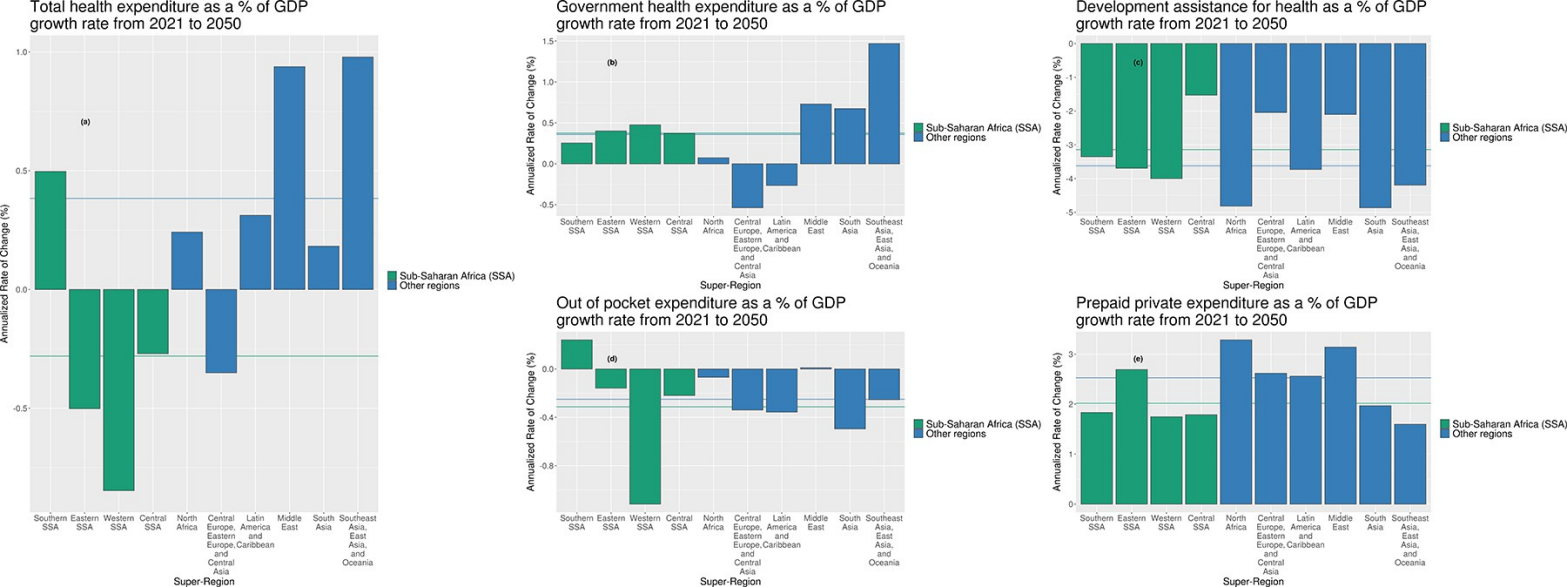
Total health spending, government health spending, development assistance for health, out-of-pocket spending and pre-paid private spending as a percentage of GDP, by super-region, 1995–2050. Excludes high-income locations. Estimates for 2022 to 2050 are forecasted.

Unlike other regions, the fraction of GDP allocated to the health sector is expected to grow slowly in sub-Saharan Africa. Fig 3(A) highlights the projected share of gross domestic product that is expected to be health spending, across regions. In all other regions of the world excluding Central Europe, Eastern Europe, and Central Asia, total health spending is expected to rise as a share of aggregate GDP, ranging from 0.2% to 1.0% annualized rate of change. However, in sub-Saharan Africa, growth in national health spending as a share of GDP in three (Eastern, Central and Western sub-Saharan Africa) out of the four sub-regions is expected to decrease. When further disaggregated by sources of health spending, government health spending (Fig 3(B) across the sub-regions in sub-Saharan Africa, measured as a fraction of GDP, is expected to grow modestly (ranging between 0.3% and 0.5%) compared to 0.1% and 1.5% across the other regions with positive growth rates. Coupled with this, DAH is not projected to grow dramatically, and in most cases is expected to shrink across all regions. Pre-paid private health spending is set to increase globally, with some regions such as eastern sub-Saharan Africa expecting 2.5% growth. In Eastern sub-Saharan Africa this growth will be fueled by countries like Kenya and Ethiopia with voluntary health insurance schemes. Lastly, out-of-pocket spending is also expected to remain largely decreasing (Fig 3C–3E).

**Fig 4:**
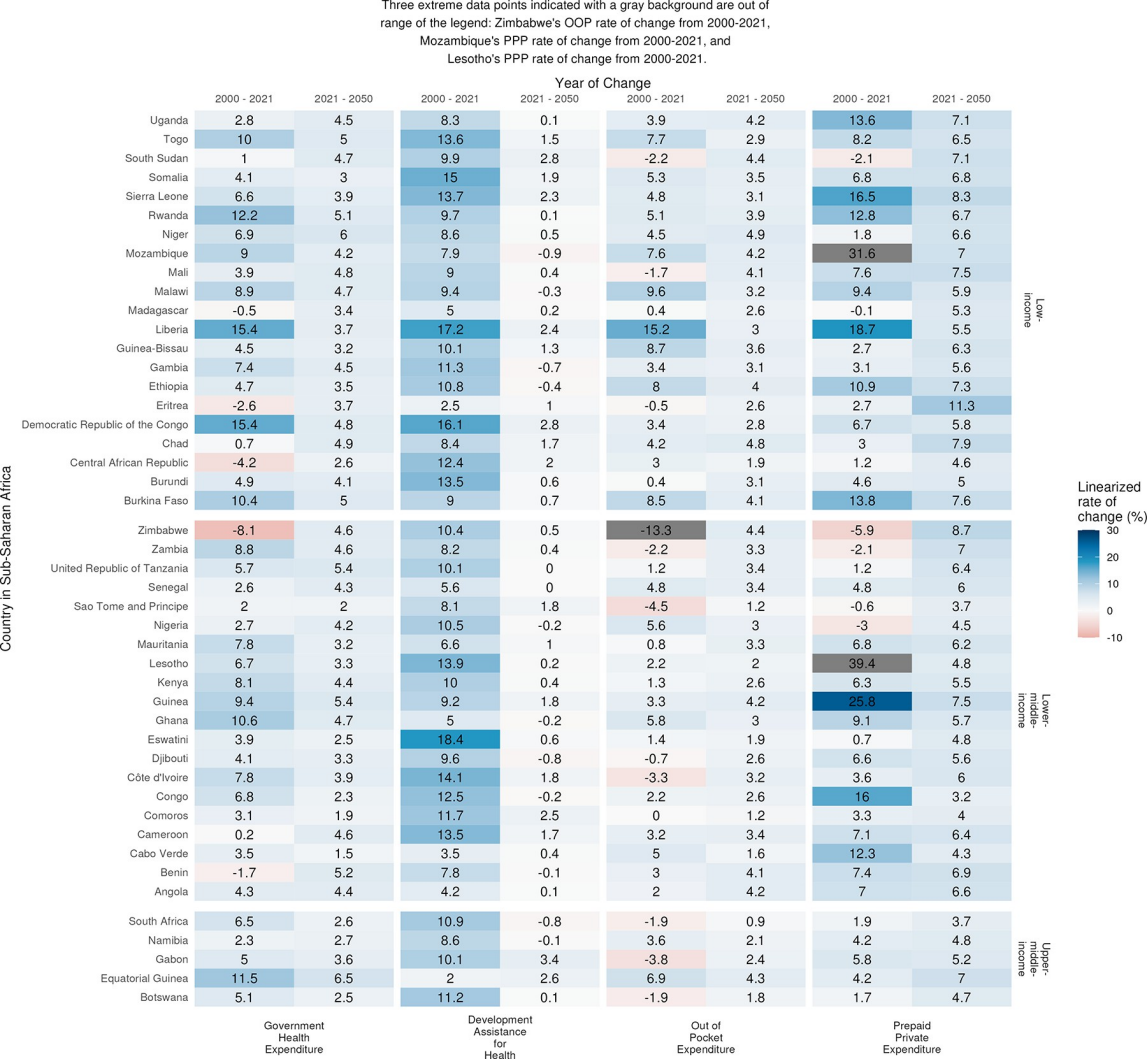
Linearized rate of change in spending by funding sources and country for 2000–2021 and 2021–2050. Sub-Saharan African countries, grouped by Work Bank income group classifications. Estimates for 2022 to 2050 are forecasted.

Within the sub-Saharan Africa region, there are also variations in financing trends. Fig 4 presents the rate of growth in government health spending, development assistance for health, out-of-pocket payment and prepaid private spending across sub-Saharan Africa countries over two specific time periods: 2000 through 2021 and 2021 through 2050. In the earlier time period (2000–2021), there were high rates of growth in development assistance for health, with 22 out of the 46 countries experiencing growth rates of above 10 percent. For government health spending, the earlier time period is a period of distinct growth with a couple countries namely Liberia, Democratic Republic of the Congo, Rwanda, Equatorial Guinea, Ghana, Burkina Faso, Togo, reporting double digit growth rates possibly due to transition into middle-income status in some countries. Most countries in lower-middle-income and upper-middle-income groups experienced a flattened or decreased trend of out-of-pocket spending in the earlier period, and a few countries namely Lesotho, Mozambique, Guinea, Liberia, Sierra Leone, Congo reported significant growth of prepaid private spending. On the contrary, the later time period (2021–2050), which projects growth rates into the future, highlight a period of lower growth, especially for government health spending and DAH, 13 countries are expected to receive less development assistance for health, across all income groups. For government spending, very moderate growth in spending is projected, with 7 countries projected to have a growth rate of more than 5% (Equatorial Guinea, Niger, United Republic of Tanzania, Guinea, Benin, Rwanda, Togo). Growth for pre-paid private spending is higher in all countries than for government health spending.

**Fig 5:**
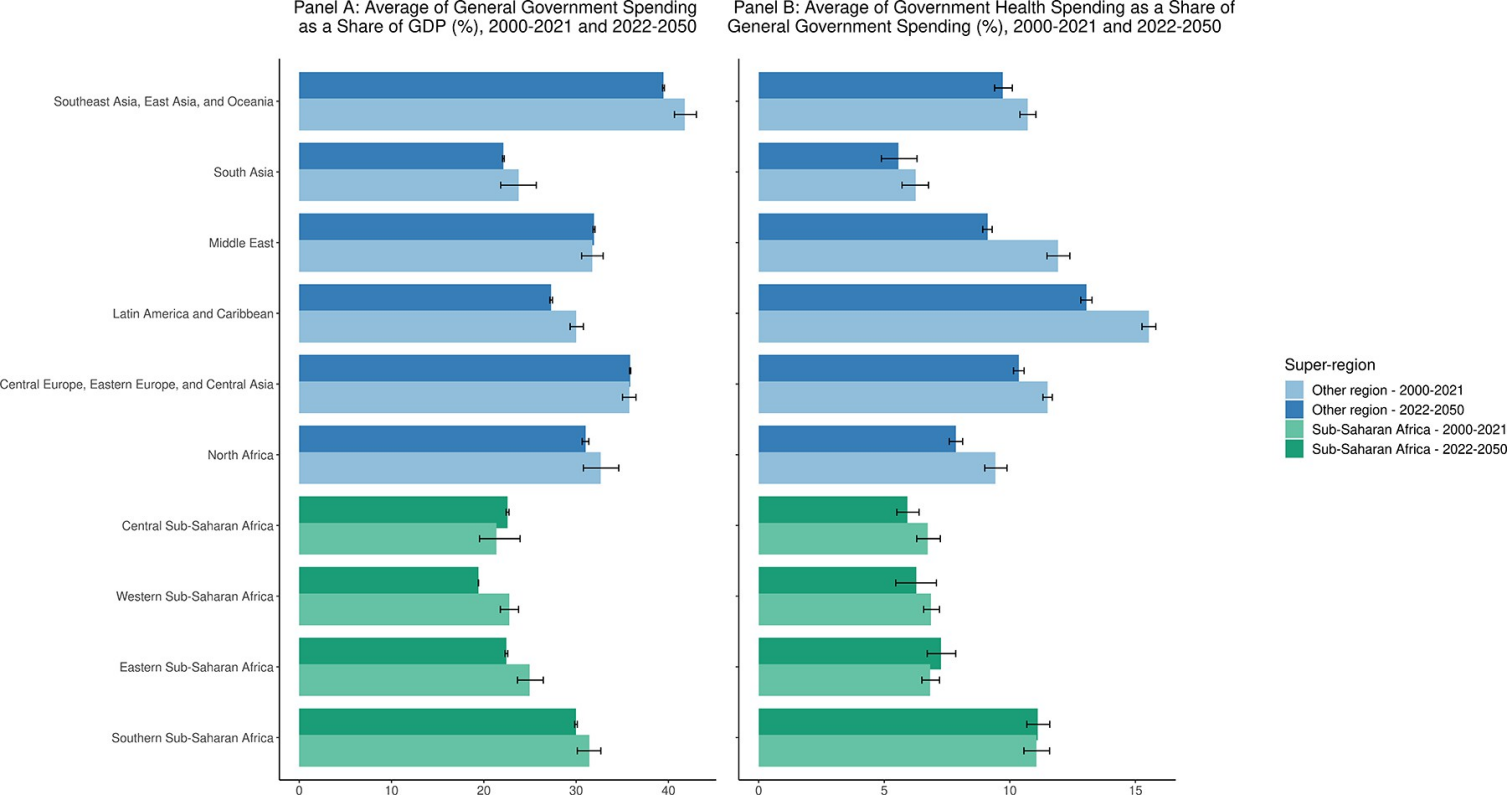
Panel A. Average of general government spending as a share of GDP, 2000–2021 and 2022–2050; Panel B. Average of government health spending as a share of general government spending, 2000–2021 and 2022–2050. Comparison of general government spending as a percent of GDP and government health spending as a percent of general government spending by region (2000–2050). Estimates for 2022 to 2050 are forecasted.

The projected modest growth in government health spending as a share of GDP seems to be driven by a relatively modest change in the share of GDP that is expected to be dedicated to health. Fig 5 panel A shows that the expected averages of the share of GDP that is general government spending across the sub-regions in sub-Saharan Africa are comparable to the expected averages in other regions. However, Fig 5 panel B, we observed that with the exception of southern sub-Saharan Africa, the expected government health spending as a share of general government spending in the sub-regions of sub-Saharan is on average lower than all other regions with the exception of South Asia.

As further shown in S1 Fig, while upper-middle-income countries will see an increasing prioritization of government spending and government health spending specifically, countries in other income-level like Uganda and South Sudan will see an increase in general government spending as a share of GDP (23·0% and 111·1% growth rate respectively) but a decrease in health spending within government health spending (36·3% and 2·1% growth rate respectively); while countries like Ethiopia will see drop in both metrics (17·4% and 36·5%).

## Discussion

This study examined health spending in sub-Saharan Africa from the past and into the future. The analyses revealed a distinct deviation in projected future health spending in sub-Saharan Africa where the growth rate of health spending as a share of national income is significantly lower relative to other regions of the world. This deviation is concerning, especially considering the global pandemic, which has made clear the need for additional investment in the health sector across all regions [17, 18]. Additionally, the study also acknowledges broader economic factors influencing this trend, emphasizing the complex landscape of health financing in the region.

One of the potential drivers of the observed measured growth in health spending in sub-Saharan Africa is the stagnation in growth of DAH. The Millennium Development Goals in the 2000s heralded a period of tremendous growth in availability of funding for health [19]. DAH grew on average 11·1% annually between 2000 and 2015, compared to recent growth rates of 4·6% each year [20]. Unlike previous epidemics, the COVID-19 pandemic was global and as such affected the economies of donor countries as well [21], raising concerns about decreases in availability of DAH funding [22] and gaps in funding for health areas where the increased support from external sources may have resulted in redirection of government funding to other areas not health-related [23, 24]. Furthermore, while DAH has shown a decreasing trend in some countries prior to the pandemic, the critical role of health investments in improving outcomes cannot be overstated. While the results may be mixed, several studies have demonstrated a positive correlation between increased health investments and improved health outcomes, particularly in low and middle-income countries, underscoring the vital importance of sustained and strategic health financing [25–27]. Additionally, for some sub-Saharan African countries such as Malawi and Somalia for whom the growth in DAH enabled it to make up the highest source of total health spending, health investments are particularly compromised as the projected growth in development assistance for health stagnates. This is because the historic large DAH investments could have had three potential pathways of impact. First, from funding increases in services that become compromised when available resources are limited. Second, by spurring a reduction in government health spending due to possible reallocation of government funding to other sectors. And lastly by prompting a shrink in total health spending as a share of GDP if DAH does not grow as fast as GDP, since it represents a substantial portion of total health spending.

While government spending on health in sub-Saharan Africa has increased in the recent past and is expected to increase into the near future, the expected rate of increase is much less than the gap to be left by the decreases in growth of DAH. Furthermore, as a share of general government spending, government spending on health is stagnating, suggesting a lower prioritization of health in the public purse. The importance of increasing government spending in the region is already acknowledged in the spirit of the Abuja declaration [28]. However, monitoring efforts such as the African Union’s Africa Scorecard for Domestic Financing of Health consistently reveal that several countries are not meeting that set target [29, 30]. The spending deficit may also jeopardize the ability of sub-Saharan African countries to meet the targets established for the heath-related Sustainable Development Goals. This projection is in consideration of the 2017 WHO estimate that indicated that for progress to be made on UHC and the other health-related targets, governments in low- and middle-income countries must spend $58 per person by 2030 in the ambitious scenario where the health-related targets are met [31, 32].

Ly and colleagues also analyze future health spending in sub-Saharan Africa [33]. Their study generates forecasted estimates of gross domestic spending that is analyzed to provide insight on future government spending on health. Like this study, they find that the expected growth in future health spending in the region is not adequate for the attainment of several of the health-related Sustainable Development Goals. For some countries, out of pocket payment and donor funding still constitute high shares of their total health spending, which compromises global health goals such as universal health coverage. They suggest a reassessment of policies and strategies targeted towards the attainment of these global health goals in the region to inspire a renewed commitment to the development of a pragmatic approach for achieving the global health goals. Results from another study by the World Bank are also aligned with the projection of limited future government health spending in sub-Saharan Africa. Specifically, the study reports that in 41 low- and lower middle-income countries government health spending is likely to remain below pre-pandemic levels until 2027 [34].

Fueled by the urgency of the pandemic, countries around the globe ramped up spending to forestall the spread of the virus and manage the economic fallout from the associated mitigation measures [17, 35]. For instance, they adopted differentiated territorial approaches, provided massive fiscal support, reallocated public funding to crisis priorities, introduced measures to support subnational finance, and announced large investment recovery packages [36, 37]. These mitigation measures exemplify the global commitment to overcoming the challenges posed by the pandemic [36, 37]. However, beyond the immediate response to COVID-19, sub-Saharan Africa confronts broader economic challenges, including political instability and inflation, further aggravated by external factors such as the Russian war. These issues complicate the economic landscape, potentially impacting health spending and the attainment of health goals in the region [9]. Additionally, in most African countries, the short-term increases in resources have been financed through borrowing, potentially constraining future public spending and specifically health sector investments. This financial strategy, while addressing immediate needs, brings forth repayment obligations that may limit future health spending. As such, there is an urgent need for innovative financing strategies to ensure fiscal health and sustainable health financing [38–40]. Domestic resource mobilization and harnessing private investment, facilitated by the development finance institutions and private sector investors, are important in this regard. These entities could support the agenda for stronger, resilient health systems through strategic investments and partnerships, aligning with global efforts to ensure sustainable health systems [40, 41].

Furthermore, in light of the growing threats from multiple epidemics, particularly in sub-Saharan Africa, there is a recognized need for additional spending on critical health infrastructure like vaccine procurement and public health surveillance systems [39]. This necessity is echoed by the G20 High Level Independent Panel on Financing the Global Commons for Pandemic Preparedness and Response, which emphasizes the need for governments in low- and middle-income countries to allocate an additional 1% of GDP annually for health spending over the next five years. This investment is crucial to build health systems capable of effectively responding to global health emergencies [39, 41]. While addressing these financing needs, it is crucial to align strategies with the broader objectives of creating health systems that are equitable and responsive to the population’s needs.

This study offers a foundational perspective on the dynamics of health financing in sub-Saharan Africa, highlighting the importance of translating these insights into tangible policy actions that can navigate the region’s complex economic and health challenges. As has been documented through the Exemplars study project, factors such as consistent stakeholder engagement, leveraging of appropriate data and evidence for decision making, integrating new initiatives into the existing health system, building research capacity and focusing on equity have been critical in countries that have achieved substantial improvements in health outcomes in recent history and as such are important factors to improve upon going forward [42].

Ultimately, while increased spending on health could be beneficial for most sub-Saharan African countries, it is imperative that countries also address issues related to the efficiency in the use of the limited resources currently available. These would include tackling challenges related to government leakages, disbursement delays and related public financial management roadblocks to harness all the available resources appropriately [43].

This study has some limitations. Firstly, its descriptive nature does not allow for causal conclusions. While the descriptive research design is well suited for the study’s objective of characterizing health spending, it does not provide definitive insight into the causes of such low health spending which may be of interest as well. Additional research studies in the future can explore the causal relationships underlying these observed patterns. Secondly, it focuses on long-term health spending projections, which, while detailed, carry inherent uncertainty and represent future possibilities rather than current realities. We have included uncertainty intervals in our reported estimates to help readers appreciate the imprecision in outcomes projected. Furthermore, to mitigate some of the concerns related to some of the underlying data in the WHO Global Health Expenditure database, we utilized qualitative scores based on the quality of the underlying estimates, to strengthen our estimates of health spending. Lastly, the study does not explore the role of private investment in health financing, an important factor in low- and middle-income countries, marking a gap in the comprehensive analysis of health spending dynamics. Despite these limitations, this study offers valuable insights into health spending trends in sub-Saharan Africa, contributing to a nuanced understanding of the region’s economic and health landscape. It lays the groundwork for further investigation into the factors influencing health financing and paves the way for more targeted, policy-oriented research, that is essential for shaping effective health systems in the region.

## Conclusion

This study reveals a concerning trend of slow growth in health sector spending in Sub-Saharan Africa, relative to GDP, from 1990 to 2050. Despite economic advancements, health financing remains stagnant, with many countries in low or lower middle-income countries facing a future of low health spending and reduced development assistance for health. This stagnation poses a significant challenge to meeting health-related Sustainable Development Goals and does not align with the African Union’s Africa Agenda 2063, which emphasizes the necessity of investing in health as a cornerstone for a prosperous future. The pandemic has further highlighted the need for global solidarity in supporting health as a global public good. Policymakers in sub-Saharan Africa must urgently address these discrepancies in health financing to not only preserve existing health gains but also to pave the way for sustainable health systems that are essential for the continent’s envisioned progress. Future research could concentrate on innovative financing strategies that can strengthen health systems in line with economic growth and the region’s broader health needs.

## Supporting information

S1 Fig Linearized rate of change in general government spending as a percentage of GDP, and government health expenditure as a percentage of general government spending, by countries, 1995–2050. Sub-Saharan African countries, grouped by World Bank income group classifications. Estimates for 2022 to 2050 are forecasted. S1 Fig available at https://cloud.ihme.washington.edu/s/5EFWzY8NXr67zeX.
(TIFF)
