# Supplementary figures and images for: Financing health in sub-Saharan Africa 1990–2050: Donor dependence and expected domestic health spending
Source: PLOS Glob Public Health. 2024 Aug 28;4(8):e0003433. doi: 10.1371/journal.pgph.0003433 (PMC11355530; doi:10.1371/journal.pgph.0003433)

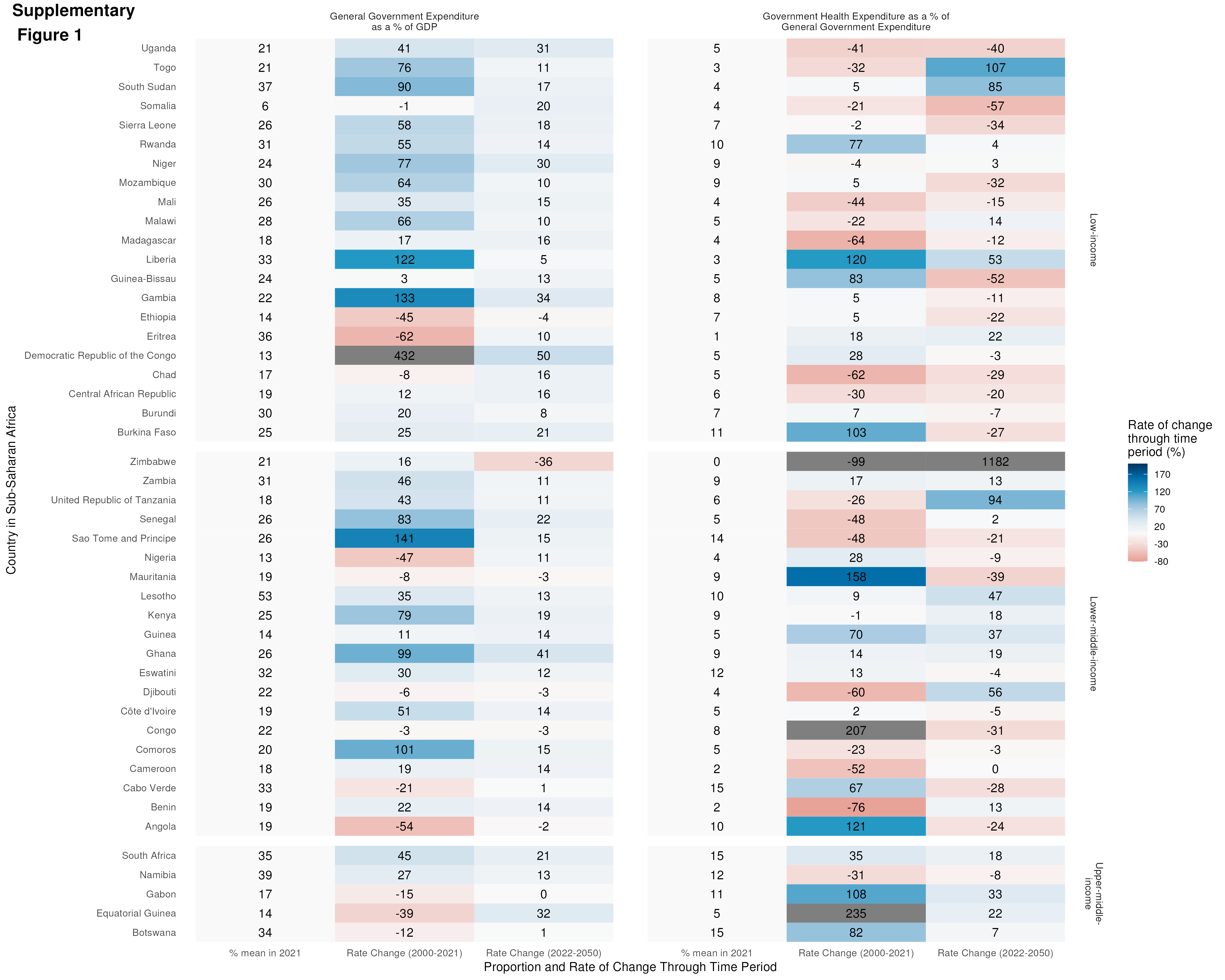

Supplement: S1 Fig — Sub-Saharan African countries, grouped by World Bank income group classifications. Estimates for 2022 to 2050 are forecasted. S1 Fig available at https://cloud.ihme.washington.edu/s/5EFWzY8NXr67zeX. (TIFF) [file pgph.0003433.s001.tiff]
